# DNA Damage Repair Gene Mutations Are Indicative of a Favorable Prognosis in Colorectal Cancer Treated With Immune Checkpoint Inhibitors

**DOI:** 10.3389/fonc.2020.549777

**Published:** 2021-02-19

**Authors:** Yipeng Song, Jian Huang, Dandan Liang, Ying Hu, Beibei Mao, Qiujing Li, Huaibo Sun, Ying Yang, Jiao Zhang, Henghui Zhang, Huan Chen, Hao Liu, Shukun Zhang

**Affiliations:** ^1^ Department of Radiation Oncology, The Affiliated Yantai Yuhuangding Hospital of Qingdao University, Yantai, China; ^2^ Institute of Oncology, First Affiliated Hospital of Kunming Medical University, Kunming, China; ^3^ Medical Department, Genecast Biotechnology Co., Ltd, Wuxi, China; ^4^ Center of Integrative Medicine, Beijing Ditan Hospital, Capital Medical University, Beijing, China; ^5^ Weihai Municipal Hospital, Cheeloo College of Medicine, Shandong University, Weihai, China; ^6^ Institute of Infectious Diseases, Beijing Ditan Hospital, Capital Medical University, Beijing, China; ^7^ Department of Oncology, Sichuan Provincial People’s Hospital, Chengdu, China

**Keywords:** DNA damage repair (DDR), immune checkpoint inhibitor (ICI), colorectal cancer (CRC), biomarker, microsatellite instability

## Abstract

**Background:**

DNA damage repair (DDR) genes were recently implicated in the anti-tumor immune response. Therefore, it is worthwhile to unravel the implications of DDR pathways in the shaping of immune responsiveness in colorectal cancer (CRC) patients receiving immune checkpoint inhibitors (ICI).

**Methods:**

We analyzed publicly available genomic data from a cohort treated with ICI from Memorial Sloan Kettering Cancer Center (MSK ICI cohort). To characterize the impact of the DDR mutation, the genomic data of The Cancer Genome Atlas (TCGA) colorectal adenocarcinoma (COADREAD) dataset was explored. We also analyzed the incidence of DDR mutation and microsatellite instability-high (MSI-H) in a Chinese CRC cohort using panel sequencing.

**Results:**

The DDR pathway was commonly mutated (21.8%) in the multicancer MSK ICI cohort, with the highest frequency of 36.4% in CRCs. Survival analysis showed that DDR mutation correlated with an improved overall survival (OS) in CRCs and pan-cancer in the MSK ICI cohort. However, no significant associations were identified in the TCGA COADREAD and MSK non-ICI CRCs. DDR mutation was associated with higher tumor mutational burden (TMB) levels and increased immune cell infiltration and immune checkpoint molecule expression in the TCGA COADREAD dataset. Last, we investigated the DDR mutational pattern and its associations with MSI-H and other genomic features in a Chinese CRC cohort. Notably, MSI-H and DDR mutation was present in 5.7% and 13.4% of cases, respectively, which suggests that DDR identifies a higher proportion of potential responders than MSI-H.

**Conclusion:**

Our data suggest that DDR mutation as an indication of enhanced cancer immunity, and it may function as a biomarker for patients with CRCs receiving ICI treatment. The high incidence of DDR mutation in the Chinese CRC cohort emphasizes the future utility of panel-based DDR evaluation in guiding ICI treatment.

## Introduction

Colorectal cancer (CRC) is one of the most common cancers in the world, and its incidence ranks third among all malignancies in men and second in women (1). Surgery is the first choice for CRC patients as a radical treatment, which has a large beneficial effect for early stage CRC. However, due to the insignificant features of CRC, most CRC patients have extensive systemic metastases at the initial diagnosis that cannot be removed by surgery, and only drug treatment remains As such, advanced CRC patients have lower survival relative to early stage CRC.

Immune checkpoint inhibitor (ICI) targeting cytotoxic T-lymphocyte–associated protein 4 (CTLA-4) or the programmed cell death protein 1 (PD-1)/programmed death-ligand 1 (PD-L1) axis were used as a recent treatment for multiple advanced solid tumors, including CRCs (2). Compared to traditional treatment, patients may achieve long-term survival and significantly reduced grade 3 or 4 adverse events after ICI treatment (3). However, the objective response rate of most cancer patients to ICI treatment is not satisfactory and treatment resistance is common (4, 5). Hyperprogression also occurred in some patients with malignant tumors (6, 7). Several candidate biomarkers may help predict the efficacy of ICI immunotherapy and identify potential responders, including PD-L1 expression (8), tumor mutational burden (TMB) (9), immune regulatory mRNA expression signatures (10), and microsatellite instability-high (MSI-H)/mismatch-repair deficiency (dMMR) (11, 12).

However, there are some limitations of these biomarkers for clinical utilization. For example, the measurement of TMB, as a continuous variable, is complicated, and it lacks standard and consistent cut-off values (13). Nivolumab plus ipilimumab provided meaningful clinical benefit in previously treated MSI-H/dMMR mCRC patients in phase II of the CheckMate-142 trial. However, no statistically significant difference in survival was observed based on the expression level of PD-L1 (14). Although MSI-H has emerged as a major predictive marker, MSI-H patients account for 15% to 20% of patients in the early stage of CRC, and only 3% to 5% in the advanced stage (15), which greatly limits the improvement of survival in microsatellite stable (MSS) or microsatellite instability-low (MSI-L) patients with advanced CRC following ICI immunotherapy. Therefore, it is of great clinical significance to identify other biomarkers to expand the population of CRC patients who may benefit from ICI.

The DNA damage repair (DDR) pathway is an important mechanism for the correction and repair of DNA damage in a timely manner to inhibit cell aging, apoptosis and carcinogenesis and ensure normal life activities (16, 17). DDR consists of eight pathways: mismatch repair (MMR), base excision repair (BER), nucleotide excision repair (NER), homologous recombination repair (HRR), nonhomologous end-joining (NHEJ), check point factors (CPF), Fanconi anemia (FA), and translesion DNA synthesis (TLS) (18). The interaction of these pathways repair DNA damage accurately and in a timely manner, prevents the occurrence of gene distortion, and ensures the integrity of the cell genome. Recent studies suggested that increasing DNA damage and reduced DNA repair abilities in cancer cells led to large aberrations in the cancer cell genomes, which distinguished these cells from normal cells and improved the effectiveness of cancer treatment (19). Wang et al. found that co-mutations in the DDR pathways of homologous recombination repair and mismatch repair (HRR-MMR) or HRR and base excision repair (HRR-BER) were potential biomarkers for ICI therapy, and these pathways were associated with increased TMB and neoantigen load and increased levels of immunity (20). However, whether DDR mutation is robustly predictive of a clinical benefit of ICI therapy for CRC patients is not clear.

Therefore, the present study investigated and discussed the correlation between DDR mutation and the efficacy of colorectal cancer immunotherapy and their effect on the molecular and immune characteristics of CRC.

## Materials and Methods

### Data Sources

The DNA sequencing data and clinical information of 1,661 patients with 10 tumor types identified using MSK-IMPACT sequencing were obtained from cBioPortal (http://www.cbioportal.org/) (21). Patients who received at least one dose of ICI treatment were selected, and patients with localized disease or who were in a trial with a data embargo were excluded. Among the 1,661 patients, we extracted genomic and clinical data of 109 CRC patients who received ICI. Complete data sets of a non-ICI-treated metastatic CRC cohort were obtained from a previous report (22) *via* cBioPortal. To unravel the mechanisms for DDR mutation in CRCs, we downloaded genomic and transcriptomic data from the TCGA (TCGA, PanCancer Atlas) *via* the cBioPortal online platform and the Genomic Data Commons (GDC) Data Portal. RNA sequencing data (FPKM) values were transformed into transcript per kilobase million (TPM) values. For each cohort, patients with complete clinical data and genomic data were included in the study.

### Definition of DNA Damage Repair and Tumor Mutational Burden

Based on the definition of the DDR pathway on the cBioPortal website, the DDR mutations were defined as any non-synonymous single nucleotide variants (SNVs), multinucleotide variants (MNVs) and short insertions and deletions (indels) in 12 genes, including checkpoint kinase 1 (CHEK1), checkpoint kinase 2 (CHEK2), RAD51 recombinase (RAD510), BRCA1 DNA repair associated (BRCA1), BRCA2 DNA repair associated (BRCA2), mutL homolog 1 (MLH1), mutS homolog 2 (MSH2), ATM serine/threonine kinase (ATM), ATR serine/threonine kinase (ATR), mediator of DNA damage checkpoint 1 (MDC1), poly(ADP-ribose) polymerase 1 (PARP1), and FA complementation group F (FANCF). According to the SNV data, the patients were divided into two groups, DDR mutation (DDR Mut) and DDR wild-type (DDR WT) subgroups.

TMB was defined as the total number of mutations divided by the number of bases in the target panel. After the cohort was sorted in ascending order, the TMB-High group was defined as the top 20% (e.g., TMB ≥ 52.66 muts/Mb in MSK ICI CRC cohort), and the TMB-Low group was defined as the bottom 80% (e.g., TMB < 52.66 muts/Mb in MSK ICI CRC cohort).

### The Colorectal Cancer Immunogram

According to a previous study, the steps of the cancer-immunity cycle are exhibited by eight axes of the immunogram score (IGS): IGS1, T cell immunity; IGS2, tumor antigenicity; IGS3, priming, and activation; IGS4, trafficking and infiltration; IGS5, recognition of tumor cells; IGS6, inhibitor cells; IGS7, checkpoint expression; and IGS8, inhibitory molecules (23). The gene sets IGS1, IGS2, IGS3, IGS4, IGS5, IGS6, IGS7, IGS8 were used in a previous study (23). Single-sample gene set enrichment analysis (ssGSEA) was used to assess the value of the immunogram scores and the relative abundance of 28 immune cell subsets. Immune cycle boxplot figures show the median immunogram scores. Two groups of immunogram radar figures show the median of ranked immunogram scores (IGSs). The tumor neoantigen value (LOH, CNV) was downloaded from the published TCGA data (24). The expression of 12 immune checkpoint negative regulators were measured as the geometric mean of gene expression in log2 of TPM+1.

### Immune Gene Signatures

The gene sets for cytolytic activity, IFN γ signature, immunocostimulators, immunoinhibitors, chemokines, T cell–inflamed gene expression profile (GEP), and MHC-class-I/II signature were defined in previous reports (25, 26). The immune gene signatures were measured as the mean value of gene expression in log2 of TPM+1.

### Assessments of DNA Damage Repair Mutations, Tumor Mutational Burden, Microsatellite Instability, and Programmed Cell Death Liagand 1 in a Chinese Colorectal CancerCohort

A total of 667 CRC subjects who were admitted to the Affiliated Yantai Yuhuangding Hospital of Qingdao University and Weihai Municipal Hospital of Shandong University from May 2018 to July 2019 were selected. The inclusion criteria for the Chinese CRC cohort were 1. primary tumor site in colon or rectum, and 2. primary tumor samples. 3. stage III/V CRCs. All patients signed an informed consent. The DDR mutation, MSI and TMB of CRC patients were detected using next generation sequencing (NGS) in Genecast Biotechnology Co., Ltd. MDC1 and FANCF were not included in the NGS-sequencing panel, and therefore, the DDR gene set in the Chinese cohort only contained 10 genes. The Genecast panel was a 1.67 Mbp-sized panel covering the exon regions of 543 genes (**Supplemental Table S1**; Genecast, Wuxi, China), including major tumor-related genes. Paired-end sequencing was performed using Illumina HiSeq X-Ten. This test identifies somatic exonic mutations using tumor-derived and matched germline normal DNA. The hg19 reference genome was used for read mapping with BWA 0.7.12 (default parameters). The expression of PD-L1 on the surface of tumor cells (TCs) and tumor-infiltrating immune cells (ICs) was assessed using IHC staining. The tissue slides were stained using an anti-PD-L1 (SP142) rabbit monoclonal primary antibody and a matched rabbit IgG-negative control.

### Statistical Analysis

The data were analyzed using R 3.6.1 and SPSS version 23.0 software. The survival analysis was performed using Kaplan-Meier curves and compared using a log-rank test. Chi-squared test or Fisher’s exact test was used to analyze the association between various genomic determinants. Student’s t-test was used to determine the differences between two groups when data were normally distributed. Otherwise, the Mann-Whitney U test was used. P<0.05 were considered statistically significant. The multivariate Cox proportional hazards regression model was performed using SPSS software.

## Results

### DNA Damage Repair Mutation Is a Pan-Cancer Prognostic Biomarker for Cancer Patients With Immune Checkpoint Inhibitor Immunotherapy

**Figure 1:**
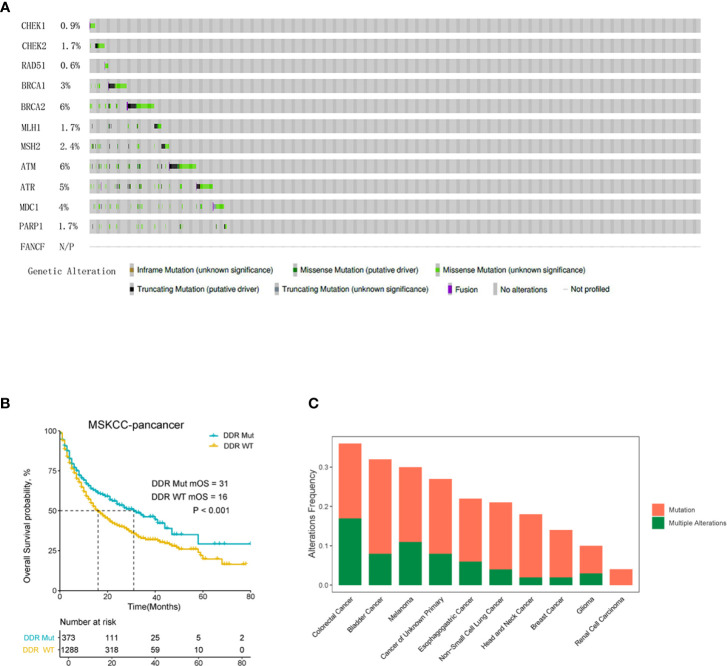
DNA damage repair (DDR) mutation is a pan-cancer prognostic biomarker for cancer patients with immune checkpoint inhibitor (ICI) immunotherapy. **(A)** The mutation frequency of 12 DDR genes in MSK-IMPACT clinical sequencing cohort was derived from cBioPortal. **(B)** Overall survival Kaplan-Meier curves of the DDR Mut and WT groups. **(C)** Frequency of DDR mutation in patients with different cancer types.

The mutation frequency of DDR genes, patient survival analysis and overall mutation of DDR genes in each tumor type in the MSK pan-cancer cohort were analyzed using the cBioPortal online platform. As shown in **Figure 1A**, the mutation frequencies of different DDR genes varied greatly. For example, the mutation frequencies of BRCA2 and ATM rose to 6%, which were significantly higher than other DDR pathway genes. The mutation frequencies of CHEK1 (0.9%), FANCF (0%) and RAD51 (0.6%) were obviously lower than the of other DDR genes. The median overall survival (OS) of the DDR Mut subgroup was significantly prolonged compared to the DDR WT group (**Figure 1B**). The mutation frequency of DDR genes in different cancer types is summarized in **Figure 1C**, and CRC patients had the highest frequency of DDR mutation, nearing 40%.

### DNA Damage Repair Mutation and Tumor Mutational Burden Correlated With the Prognosis of Colorectal Cancer Patients With Immune Checkpoint Inhibitor Immunotherapy

**Figure 2:**
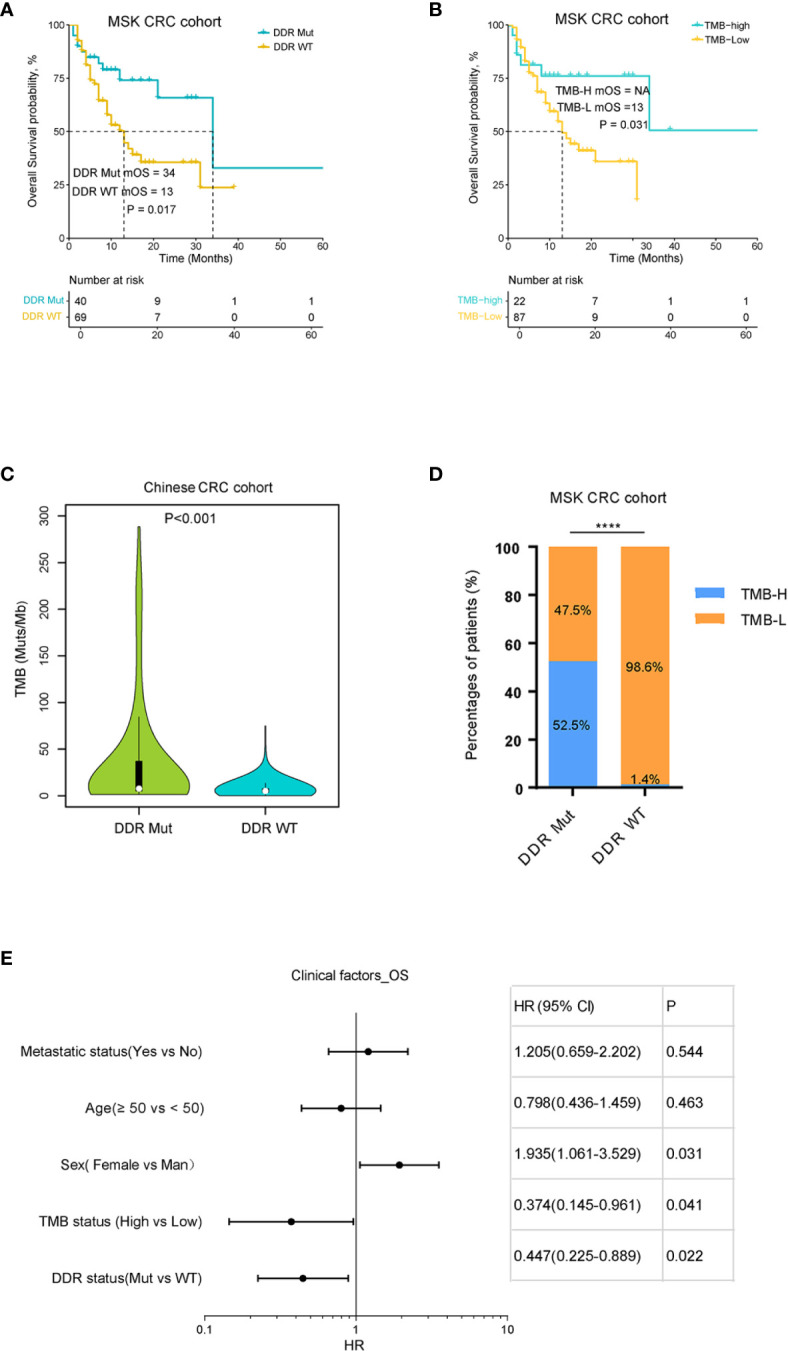
DNA damage repair (DDR) mutation and tumor mutational burden (TMB) correlated with the prognosis of colorectal cancer (CRC) patients with immune checkpoint inhibitor (ICI) immunotherapy. **(A)** Kaplan-Meier curves of overall survival for DDR Mut and WT groups. **(B)** Kaplan-Meier curves of overall survival for TMB-High and TMB-Low groups. **(C)** Distribution of TMB in DDR Mut and WT groups. **(D)** The proportion of TMB-High and TMB-Low in DDR Mut and WT groups. **(E)** Univariate Cox regression analysis of overall survival (Cox proportional hazards regression model). ****P < 0.0001.

To investigate the impact of DDR mutation on ICI immunotherapy in CRCs, we analyzed the genomic and clinical data of CRC patients treated with ICI provided by MSK. The results showed that patients with DDR mutation had a significantly improved OS compared to the DDR wild-type population (P=0.016) (**Figure 2A**). We also evaluated the influence of TMB on OS in CRC patients treated with ICI therapy. As shown in **Figure 2B**, there was a statistically significant difference in the OS curve between TMB-H (top 20%) and TMB-L subgroups (bottom 80%), which is consistent with a previous report (21). We also examined the correlation between TMB and DDR mutation, which revealed that the proportion of TMB-H in the DDR Mut subgroup was substantially higher than the DDR WT group (52.5% vs. 1.4%, P<0.001) (**Figures 2C–D**). Although these observations suggest a redundant predictive role for DDR mutation in the TMB-H CRCs, we presented novel evidence that DDR mutation (40 of 109 CRCs) may help identify more potential responders to ICI relative to TMB-H (20% of CRCs). In the TMB-low subgroup (80% of CRCs), DDR mutated CRCs revealed an improved OS when compared with the WT subgroup, with a certain trend toward significance (P=0.084) (**Supplemental Figure S1A**). In univariate Cox regression analysis, the following parameters significantly affected overall survival (P<0.05): DDR status, TMB status, sex (**Figure 2E**). When these factors were included in multivariate analysis, sex remained an independent prognostic factor (P=0.042), TMB and DDR mutation may not function independently (P=0.211, P=0.238) (**Supplemental Figure S1B**).

### Impact of DNA Damage Repair Mutation on Non-Immune Checkpoint Inhibitor-Treated Colorectal Cancers

**Figure 3:**
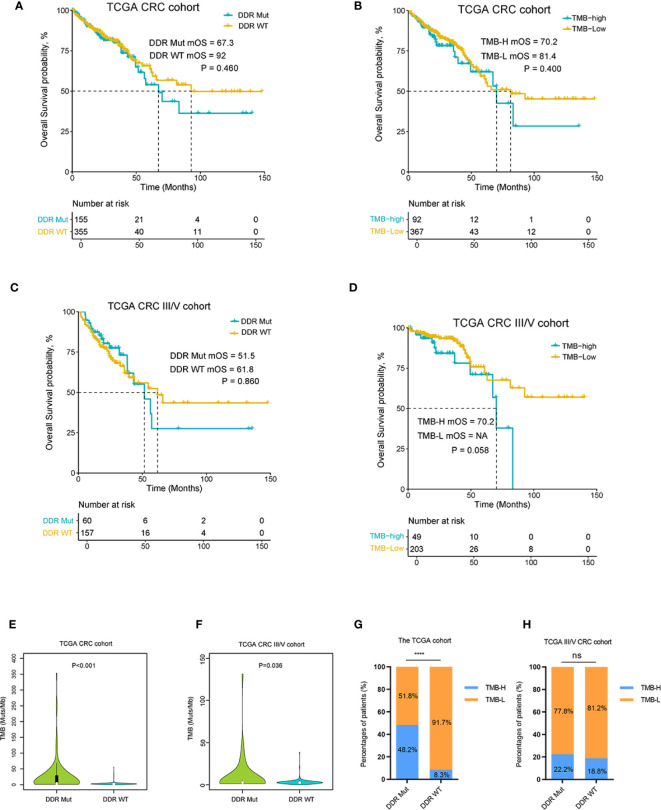
DNA damage repair (DDR) mutation may be a specific biomarker to predict the efficacy of immune checkpoint inhibitor (ICI) immunotherapy in colorectal cancer (CRC). **(A)** Kaplan-Meier curves of overall survival in the The Cancer Genome Atlas (TCGA) CRC cohort and **(B)** the TCGA III/V CRC cohort for DDR Mut and WT groups. **(C)** Kaplan-Meier curves of overall survival in the TCGA CRC cohort and **(D)** the TCGA III/V CRC cohort for the TMB-High and TMB-Low groups. **(E, F)** Distribution of TMB in the DDR Mut and WT groups **(E)** in the TCGA CRC cohort and **(F)** the TCGA III/V CRC cohort. **(G, H)** The proportion of TMB-High and TMB-Low in DDR Mut and WT groups **(G)** in the TCGA CRC cohort and **(H)** the TCGA III/V CRC cohort. ****P < 0.0001, ns, no significant difference.

To examine the effect of DDR mutation on CRC patients with traditional treatment modalities, we analyzed DDR mutation and TMB values for an association with patient prognosis in a TCGA and another MSK non-ICI cohort (22). As shown in **Figures 3A–D** and **Supplemental Figure S2A**, there was no significant difference in OS curves between the DDR Mut and WT groups in the TCGA CRC cohort (P=0.460), the TCGA CRC III/V cohort (P=0.860) or the MSK non-ICI CRCs (P=0.630). We also did not observe any differences in clinical outcomes in these cohorts when stratified by TMB (P*>*0.05 for all comparisons). We investigated the association between TMB and the incidence of DDR mutation in the TCGA CRC cohort, which showed that the proportion of TMB-H in the DDR Mut group was notably higher than the DDR WT group (P<0.001) (**Figures 3E, G**). Notably, although there was an association between the TMB value and the incidence of DDR mutation in the TCGA CRC III/V subset (**Figure 3F**, P=0.036), the proportion of TMB-H in the DDR Mut and WT subgroups was not significantly different (P=0.360) (**Figure 3H**). We also examined the association between MSI status and DDR mutational status in the TCGA CRC cohort and the III/V CRC subset, which showed that the proportion of MSI-H in the DDR Mut group was markedly higher than the DDR WT group (P<0.001) (**Supplemental Figures S2B, S2C**).

### Association Between DNA Damage Repair Mutation and Immune Microenvironment

**Figure 4:**
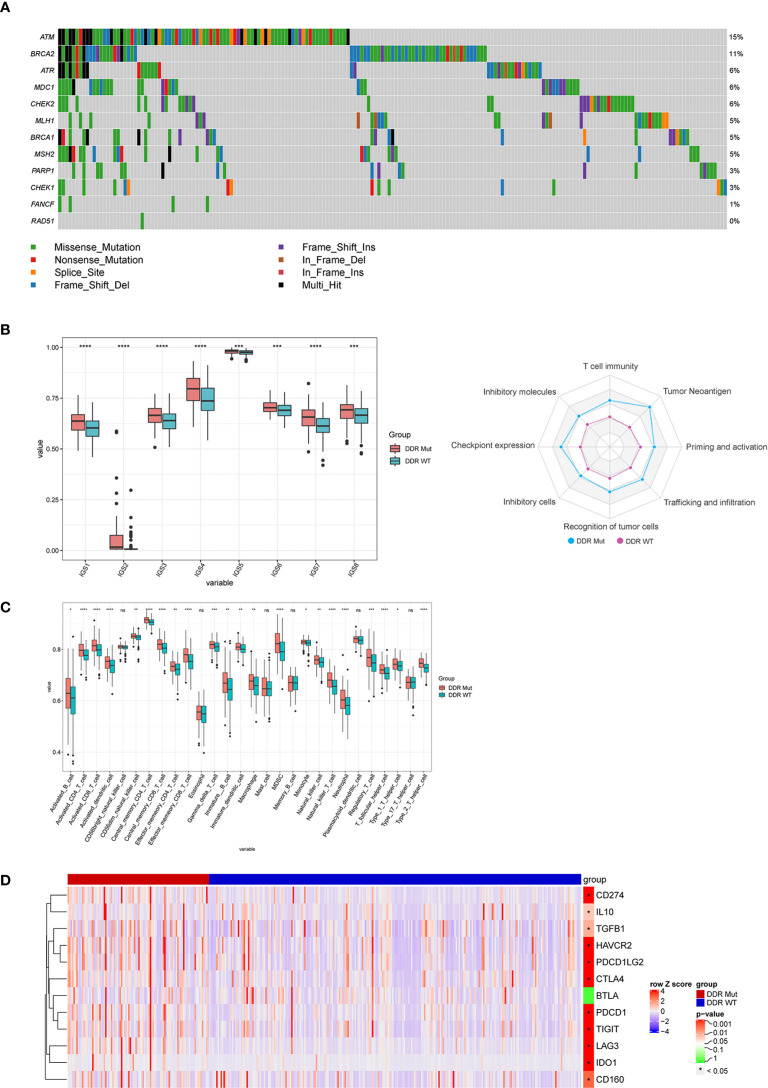
DNA damage repair (DDR) mutation improves the efficacy of immune checkpoint inhibitor (ICI) immunotherapy *via* regulation of the immune microenvironment. **(A)** The mutation frequency of 12 DDR genes in the The Cancer Genome Atlas (TCGA) colorectal cancer cohort was derived from the TCGA website. **(B–D)** Comparison of **(B)** immune cycle, **(C)** 28 immune cell subsets, and **(D)** 12 immune checkpoint negative regulators between the DDR Mut and WT groups. ns P>0.05, *P<0.05, **P<0.01, ***P<0.001, ****P<0.0001.

To explore the associations of DDR mutations with immunotherapy signatures, we analyzed the genomic and transcriptomic data of the TCGA COADREAD data set. First, the mutational pattern of DDR genes in the TCGA CRC patients was consistent with that of the MSK CRC cohorts receiving ICI (**Figure 4A**). As expected, BRCA2 and ATM were among the top mutated DDR genes (**Figure 4A**). Next, we examined the immunological features between DDR Mut and WT CRCs because previous studies found that the efficacy to ICI was related to the activity of the immune microenvironment (27, 28). Notably, we observed a substantial enhancement of all immunity cycles in the DDR Mut subgroup versus the DDR WT CRCs (**Figure 4B**). Infiltrating immune cells (**Figure 4D**), T cell–inflamed GEP and IFN-γ were also significantly increased in patients with DDR mutation (**Supplemental Figure S3A-B**), and the copy number variation (CNV) and loss of heterozygosity (LOH) were obviously decreased (**Supplemental Figure S3C-D**). Mutation in the DDR pathway was associated with an evident enrichment of multiple immune signatures, including chemokines, cytolytic makers, MHC-class II molecules, immunostimulators, and immunoinhibitors. We observed elevated expression of 11 immune checkpoint regulators in the DDR Mut subgroups, including PD-L1 (CD274), CTLA4, PD1 (PDCD1), PD-L2 (PDCD1LG2), lymphocyte activating 3 (LAG3), hepatitis A virus cellular receptor 2 (HAVCR2), T cell immunoreceptor with Ig and ITIM domains (TIGIT), and indoleamine 2,3-dioxygenase 1 (IDO1) (P<0.001 for all comparisons, **Figure 4D** and **Supplemental Figure S4**). To explore the effects of MSI-H in DDR mut group, we analyzed infiltrating immune cells and immunotherapy signatures of the MSI-H and MSS subgroup, respectively. We observed infiltrating immune cells were also significantly increased in patients with MSI-H (**Supplemental Figure S5A**), the trend of the differences in immune cell subsets between DDR status subgroup and MSI status subgroup was consistent. In MSI-H subgroup, there was no significantly difference in immune cell subsets between DDR mut and wild type group (**Supplemental Figure S5B**). But, in MSS subgroup, five infiltrating immune cells (Activated_dendritic_cell, CD56 bright_natural_killer_cell, Effector_memeory_CD4_T_cell, Immature_dendritic_cell, Type_2_T_helper_cell) were also significantly increased in patients with DDR mut (**Supplemental Figure S5C**). Specifically, NK cells are a group of innate cytolytic effector cells that participate in immune surveillance, and NK infiltration in tumors has been associated with an improved prognosis for cancer patients (29–31). In addition, we observed the copy number variation (CNV) were obviously decreased (**Supplemental Figure S5D-E**) in patients with DDR mutation, either in MSI-H or MSS subgroups. A lower burden of copy-number loss (CNloss) was observed in responders to ICB treatment in melanoma (32). According to Zhihao Lu, a lower copy-number alteration (CNA) burden may correlate with an activated inflammatory response in the TME. CNA-low GC and CRC samples were infiltrated with diverse immune cell types, including activated CD8+ T cells, activated CD4+ T cells, natural killer (NK) cells, and NK T cells (33). Taken together, our findings indicate that DDR mutation can select out MSS patients with rich immune infiltration. But how DDR interplays with MSI-H or TMB-high is needed to further explore.

### Landscape of DNA Damage Repair Gene Mutation in a Chinese Colorectal Cancer Cohort

**Figure 5:**
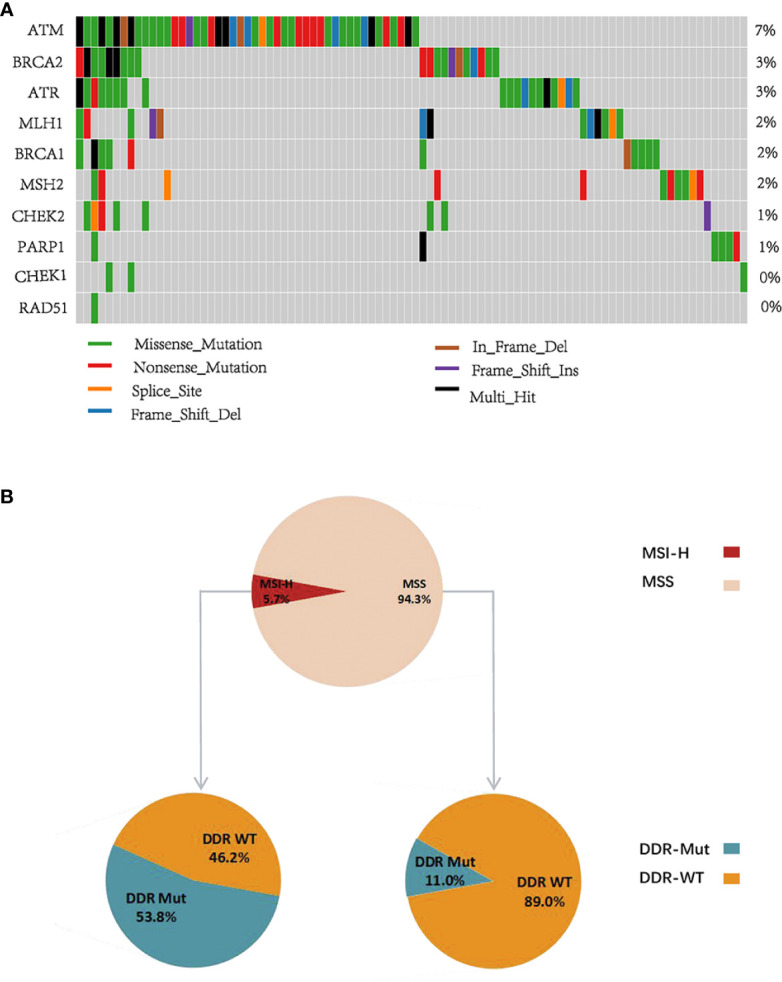
DNA damage repair (DDR) gene mutational pattern in a Chinese colorectal cancer (CRC) cohort. **(A)** The mutation frequency of 10 DDR genes in Chinese CRC patients. **(B)** The proportion of DDR Mut and DDR WT in the microsatellite instability-high (MSI-H) and microsatellite stable (MSS) groups.

Because of the potential predictive value of DDR mutation in guiding patient selection in CRCs, we further examined the mutational status of DDR genes in a Chinese CRC cohort. Our data revealed that the mutation frequency of ATM was 7%, which was significantly higher than the other DDR genes, and the mutation frequencies of CHEK1 and RAD51 were notably lower than the other DDR genes (**Figure 5A**). Notably, 5.7% of Chinese CRC patients had MSI-H, and the incidence of DDR mutation was 13.8%. We further identified that the proportion of patients with DDR mutation in the MSI-H group was 53.8% and 11% in the MSS group (**Figure 5B**), which suggests that DDR mutation expands the benefit to the subgroup receiving ICI treatment, especially MSS CRCs. Last, we studied the association between these genomic determinants and PD-L1. Notably, the percentages of MSI-H, TMB-H, and PD-L1-positive CRCs in the DDR Mut subgroup were remarkably higher than the DDR WT group (**Supplemental Figure S6A–D**, P<0.001 for all comparisons). Collectively, these observations indicated that DDR mutation may represent an intrinsic pathological feature of tumor cells and the immune microenvironment.

## Discussion

Immune checkpoints are sites on the surface of T cells that are involved in anti-tumor immunity and combine with corresponding ligands on the surface of tumor cells or antigen-presenting cells to suppress the immune response, which leads to the escape of tumor cells (34). ICI improve the effects of anti-tumor immunotherapy by blocking the immunosuppressive effect of PD-1/PD-L1, CTLA-4, and other immune checkpoints. ICI therapy demonstrated impressive clinical efficacy in the treatment of multiple advanced cancers. However, it also has some disadvantages, such as a low objective response rate, high price, and the possibility of immune inflammatory or tumor hyperprogression (4–7). Some biomarkers may help predict the efficacy of ICI immunotherapy and maximize the benefit to patients and avoid treatment risks. For example, the expression of PD-L1 in tumor tissues is an indicator that predicts the effectiveness of PD-1**/**PD-L1 inhibitors, and it is a standard predictive biomarker for the first-line treatment pembrolizumab (8). TMB and MSI are also important biomarkers to predict the response to ICI. When tumor tissue is in a TMB-H or MSI-H state, the effective rate of ICI therapy is higher than for tissues in TMB-L or MSI-L/MSS states (9, 11, 12). However, these biomarkers have limitations, which highlight the clinical need to identify better predictive biomarkers.

The DDR pathway plays an important role in maintaining the normal life activities of cells (35). During the early stage of cancer initiation, the DDR pathway accurately and timely repairs mutated genes and hinders the development of tumors. However, as cancers develop, the DDR pathway also repairs the chemotherapy- or radiotherapy-induced DNA damage in cancer cells, which results in therapy resistance. If the DDR pathway is disrupted, the frequency of genomic aberrations in tumor cells increases dramatically, and abnormal proteins produced by mutated genes increase accordingly (19, 30). These abnormal proteins are more likely to act as antigens to activate the human immune system, which increases the probability of the patient benefiting from ICI immunotherapy (19). However, to our knowledge, the predictive value of DDR mutation for ICI treatment efficacy in CRC has not been reported, and it is worth clarifying.

The present study showed that DDR mutation was associated with a favorable median OS in CRC patients treated with ICI (**Figure 2A**), but no significant difference was identified in the prognosis of patients with or without DDR mutation with conventional treatment (**Figures 3A, C**). Similar findings were obtained for TMB and patient clinical outcomes (**Figures 2B** and **3B, D**). These observations indicated that DDR mutation may be a specific biomarker to predict the efficacy of ICI immunotherapy in CRCs and may complement TMB as a biomarker. Wang et al. speculated that high TMB were the result of increasing DDR mutatio-n accumulation in clinical NSCLC and melanoma cohorts (20). Therefore, we determined the correlation between DDR mutation and TMB values. The proportion of TMB-H in CRC patients with DDR mutation was substantially higher than patients without DDR mutation in all CRC cohorts (**Figures 2**, **3** and **Supplemental Figure S5**). In a Chinese CRC patients undergoing conventional treatments, we identified that 53.8% of patients with MSI-H had a DDR mutation and 11% of the MSS population also had a DDR mutation, which suggests that DDR mutations would help identify a population of potential responders to ICI in the MSS subgroup (**Figure 5B**). However, further cohort studies performed in the MSS/MSI-L CRCs receiving ICI would be helpful to confirm the predictive role of DDR mutation.

We further investigated the mutation frequency in the DDR pathway and found that the incidence of mutations of ATM and BRCA2 in the DDR pathway were significantly higher than for other genes, which is consistent with the finding that melanoma patients who responded to ICI generally harbor mutations in BRCA2 (36). Some reports also confirmed a close relationship between DDR mutation and the sensitivity to platinum neoadjuvant chemotherapy and chemoradiotherapy, including ERCC2, ATM, FANCD2, PALB2, BRCA1, BRCA2, and RB1 (37, 38). Other DDR genes were mutated with different mutation frequencies, which may also affect the response to ICI. However, due to the lack of research on DDR genes and the certain interaction between different DDR pathways, further in-depth studies are needed.

The tumor immune microenvironment includes anti-tumor immune effector cells, immunosuppressive cells and immune signaling molecules, which play important roles in tumor development and clinical treatment. Previous studies demonstrated that the intrinsic responsiveness of CRCs was closely linked to the immune microenvironment (27, 28). As previously reported, anti-cancer immunity is a dynamic process that is described as a cancer immunity cycle of eight steps: 1, T-cell immunity; 2, tumor antigenicity; 3, priming and activation; 4, trafficking and infiltration; 5, recognition of tumor cells; 6, absence of inhibitory cells; 7, absence of checkpoint expressions; and 8, absence of inhibitory molecules (23, 39). Notably, we presented a novel finding that DDR mutation was associated with a substantial enhancement in all steps of the cancer immunity cycle (**Figure 4B**). The ssGSEA analysis also revealed that a number of immune cells and 11 immune checkpoint molecules were enriched in CRCs with DDR mutation compared to the patients without DDR mutation (**Figures 4C, D** and **Supplemental Figure S4**). Our data suggest that DDR mutation results in the activation of cytotoxic T cells *via* the upregulation of immune checkpoint expression, which promotes the responsiveness to ICI. Previous studies also demonstrated the interaction of DDR with the immune system, immune signatures, and immune-related genes (35). For example, BRCA1/2-mutated ovarian tumors showed increased expression levels of PD-1/PD-L1 and tumor-infiltrating lymphocytes (TIL), such as CD3+ and CD8+ TILs (40, 41). Therefore, it would be of great interest to clarify the underlying mechanisms of the interaction between DDR mutation and microenvironment remodeling across different cancer types.

Base on contemporary meta-analysis of all available immunotherapy clinical trials, the association between patient sex with immunotherapy efficacy and OS was controversial (42, 43). Although, in our study the difference in efficacy between men and female treated with immune checkpoint inhibitors was significant (**Supplemental Figure S1B**), but our cohort study was prone to bias of sex and small sample size. Future research should guarantee greater inclusion of female in trials and focus on improving the effectiveness of immunotherapies in female, perhaps exploring different immunotherapeutic approaches in men and female. The multivariate cox model result revealed that both TMB and DDR mutation may do not function independently (**Supplemental Figure S1B**), and showed each feature as trending significant, suggesting the interplay among these factors. Future research should guarantee greater sample size in trials to explore how DDR interplays with other clinical factors. DDR mutation might be one of many predictive biomarkers in CRCs, as previous findings have shown the prognostic and prognostic significance of MSI-H, TMB, and PD-L1 (8–12). Therefore, DDR along with other factors should be taken into account during clinic utilization.

In conclusion, DDR mutation may be a candidate predictive biomarker for CRC patients receiving ICI treatments. DDR mutation is associated with enriched immune cell infiltration, enhancement of the cancer immunity cycle, elevated TMB, and abundant immune checkpoint expression in the tumor microenvironment.

## Data Availability Statement

Publicly available datasets were analyzed in this study. These data can be found here: cBioPortal (http://www.cbioportal.org/).

## Author Contributions

YS, JH, DL, and YH conceived and designed this study. BM, QL, HS, YY, and JZ analyzed the data and interpreted the results. YS, JH, DL, and HZ drafted the manuscript. HC, HL, and SZ provided critical comments, suggestions, and revised the manuscript. All authors read and approved the final version of the manuscript. All authors contributed to the article and approved the submitted version.

## Funding

This work was supported by grants from the General Project of Yunnan Provincial Science and Technology Department (2019FB114).

## Conflict of Interest

Authors DL, BM, HS, YY, JZ, HC were employed by company Genecast Biotechnology Co., Ltd.

The remaining authors declare that the research was conducted in the absence of any commercial or financial relationships that could be construed as a potential conflict of interest.

## Supplementary Material

Supplementary Figure 1 Association between DDR mutation and the clinic outcome of CRC patients with ICI immunotherapy in the TMB-low subgroup. **(A)** Kaplan-Meier curves of overall survival for DDR Mut and WT groups. **(B)** Multivariable Cox regression analysis of overall survival (Cox proportional hazards regression model).

Supplementary Figure 2 The association between DDR mutation status and patient prognosis in the data of Metastatic Colorectal Cancer (MSKCC), DDR mutation status and MSI in the TCGA CRC cohort. **(A)** Kaplan-Meier curves of overall survival in the Metastatic Colorectal Cancer (MSKCC, Cancer Cell 2018). **(B, C)** The proportion of DDR Mut and DDR WT in the MSI-H and MSS groups of the TCGA CRC cohort and III/V cohort.

Supplementary Figure 3 The association between DDR mutation and tumor immune features in the TCGA CRC cohort. **(A–D)** Comparison of **(A)** GEP, **(B)** IFN γ, **(C)** LOH, and **(D)** CNV between the DDR Mut and WT groups. **(E)** Comparison of 11 tumor immune features between the DDR Mut and DDR groups. ns P>0.05, * P<0.05, ** P<0.01, ***P<0.001.

Supplementary Figure 4 The association between DDR mutation and immune checkpoint negative regulators in the TCGA CRC cohort. Comparison of 8 immune checkpoint negative regulators between the DDR Mut and DDR groups. ***P<0.001, ****P<0.0001.

Supplementary Figure 5 DDR mutation improves the efficacy of ICI immunotherapy *via* regulation of the immune microenvironment in MSS subgroup. **(A)** Comparison of 28 immune cell subsets between the MSI-H and MSS groups. **(B)** Comparison of 28 immune cell subsets, **(D)** LOH and CNV between DDR Mut and WT groups in MSI-H subgroup. **(C)** Comparison of 28 immune cell subsets, **(E)** LOH and CNV between DDR Mut and WT groups in MSS subgroup. ns P>0.05, * P<0.05, ** P<0.01, ***P<0.001, ****P<0.0001.

Supplementary Figure 6 The association between the TMB value and the incidence of DDR mutation, the MSI status and the incidence of DDR mutation, PD-L1-positivity and the incidence of DDR mutation in a Chinese CRC cohort. **(A)** Distribution of TMB in the DDR Mut and WT groups. **(B)** The proportion of TMB-High and TMB-Low in the DDR Mut and WT groups. **(C)** The proportion of MSI-H and MSS in the DDR Mut and WT groups. **(D)** The proportion of PD-L1+ and PD-L1- in the DDR Mut and WT groups.
