# Supplementary figures and images for: DNA Damage Repair Gene Mutations Are Indicative of a Favorable Prognosis in Colorectal Cancer Treated With Immune Checkpoint Inhibitors
Source: Front Oncol. 2021 Feb 19;10:549777. doi: 10.3389/fonc.2020.549777 (PMC7934780; doi:10.3389/fonc.2020.549777)

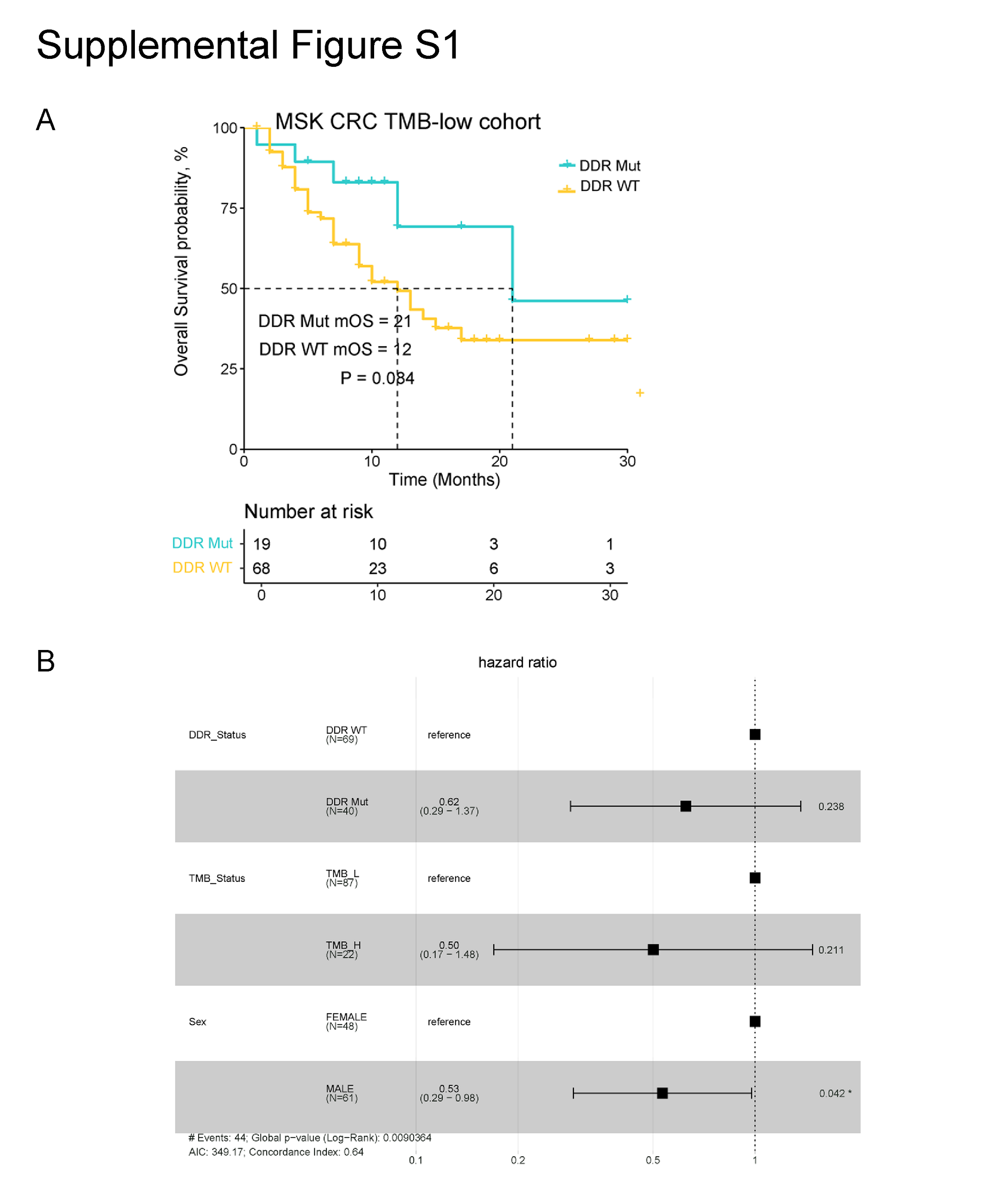

Supplement: Supplementary Figure 1 — Association between DDR mutation and the clinic outcome of CRC patients with ICI immunotherapy in the TMB-low subgroup. (A) Kaplan-Meier curves of overall survival for DDR Mut and WT groups. (B) Multivariable Cox regression analysis of overall survival (Cox proportional hazards regression model). [file Image_1.tif]

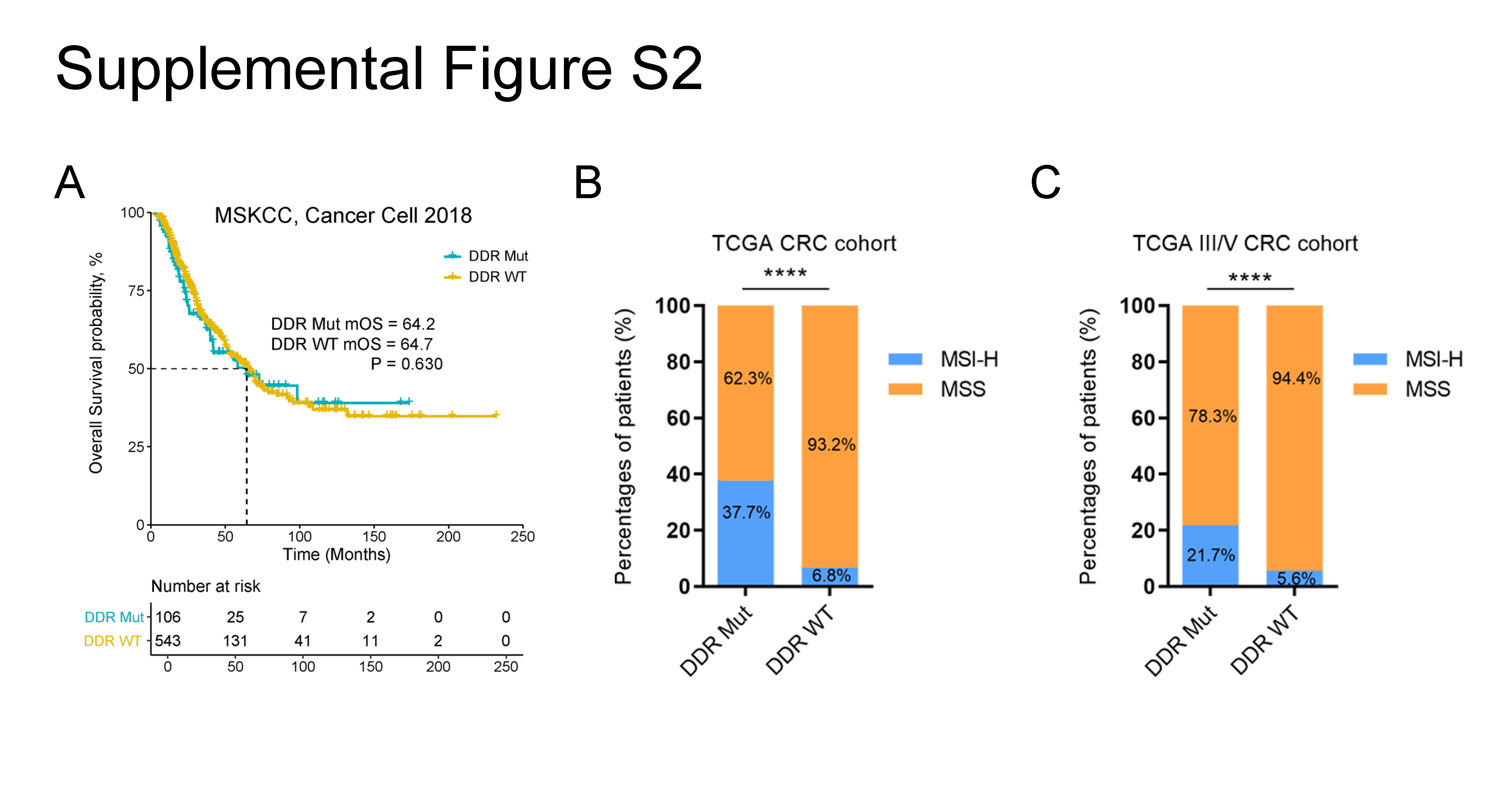

Supplement: Supplementary Figure 2 — The association between DDR mutation status and patient prognosis in the data of Metastatic Colorectal Cancer (MSKCC), DDR mutation status and MSI in the TCGA CRC cohort. (A) Kaplan-Meier curves of overall survival in the Metastatic Colorectal Cancer (MSKCC, Cancer Cell 2018). (B, C) The proportion of DDR Mut and DDR WT in the MSI-H and MSS groups of the TCGA CRC cohort and III/V cohort. [file Image_2.tiff]

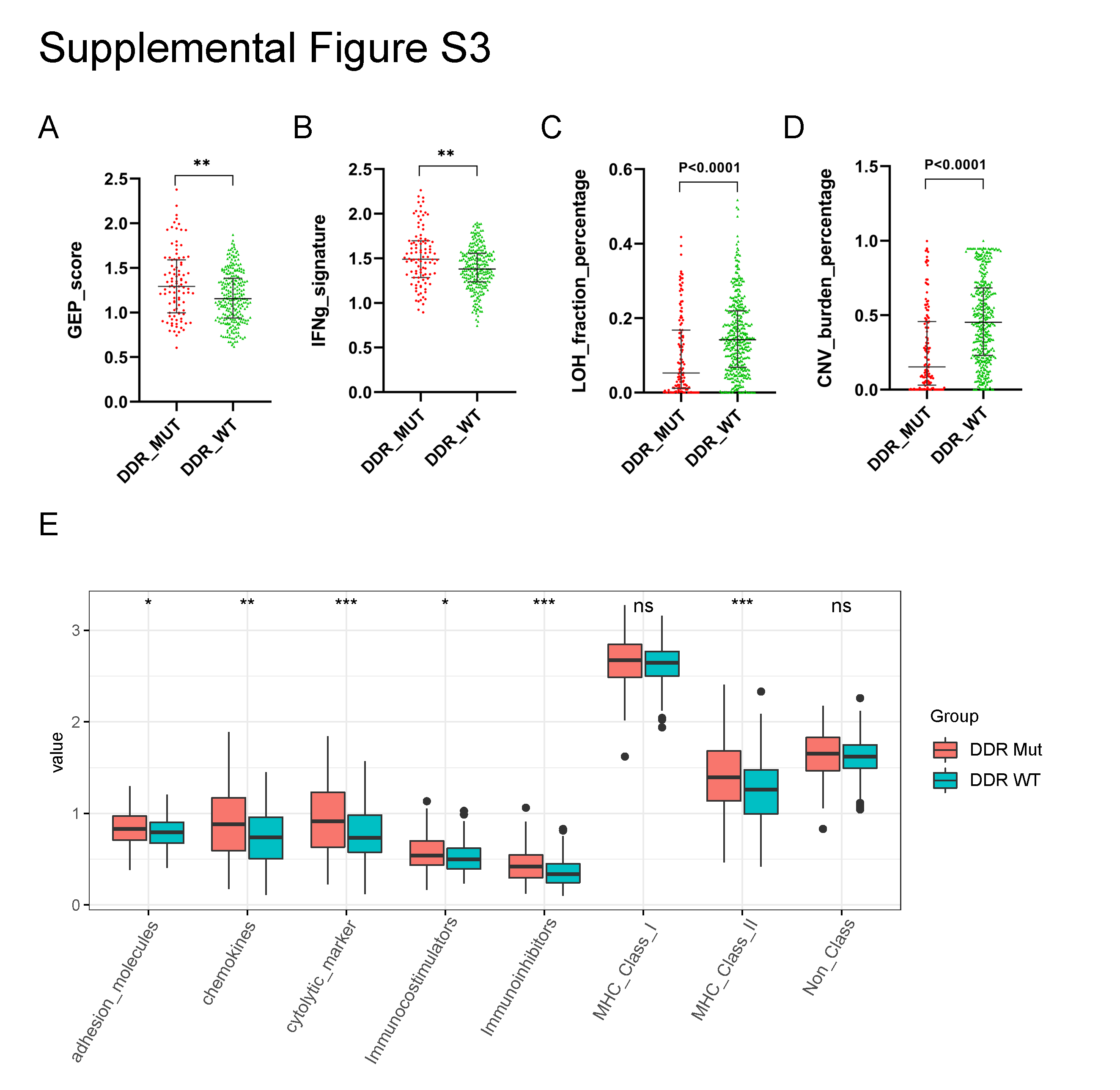

Supplement: Supplementary Figure 3 — The association between DDR mutation and tumor immune features in the TCGA CRC cohort. (A–D) Comparison of (A) GEP, (B) IFN γ, (C) LOH, and (D) CNV between the DDR Mut and WT groups. (E) Comparison of 11 tumor immune features between the DDR Mut and DDR groups. ns P>0.05, * P<0.05, ** P<0.01, ***P<0.001. [file Image_3.tif]

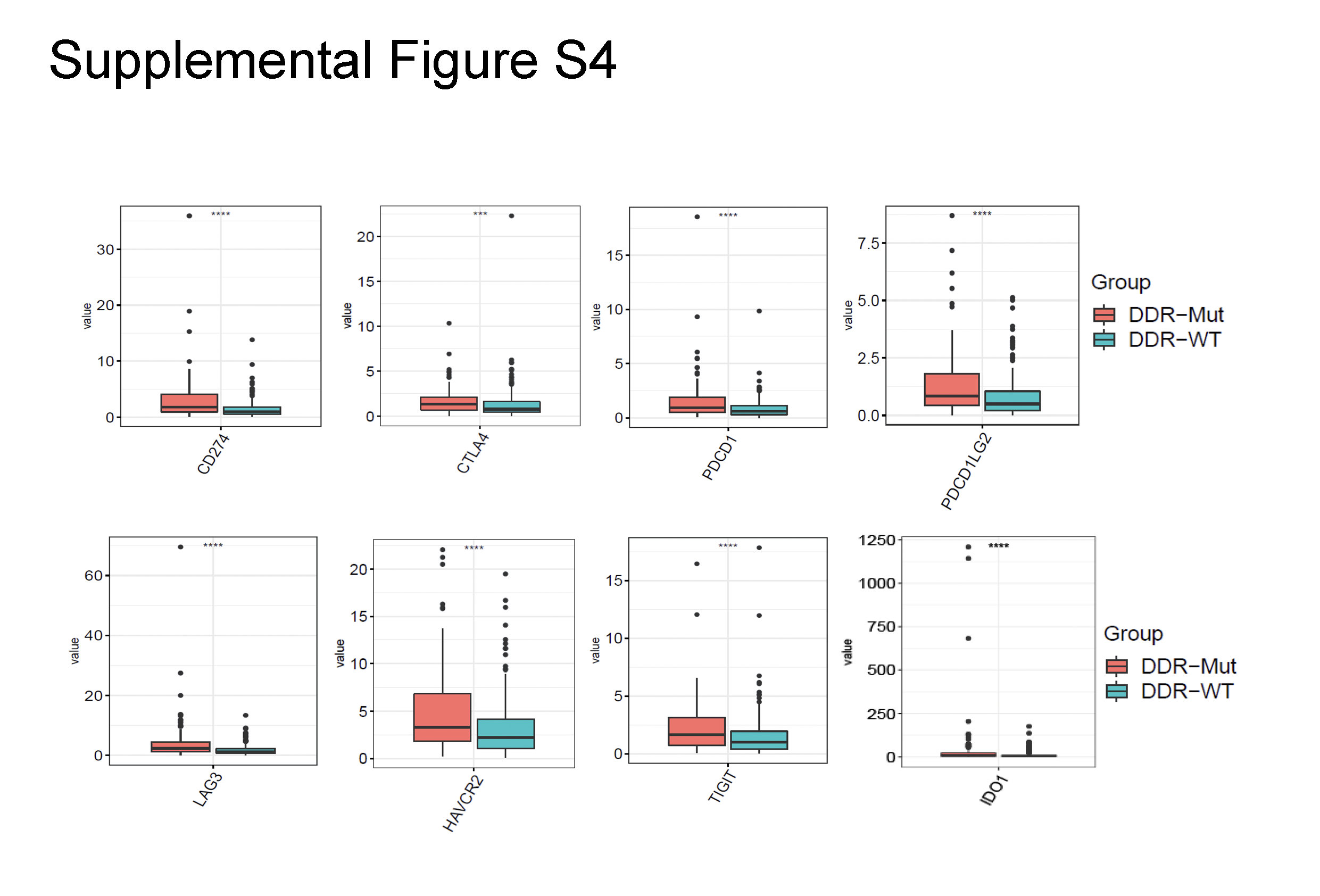

Supplement: Supplementary Figure 4 — The association between DDR mutation and immune checkpoint negative regulators in the TCGA CRC cohort. Comparison of 8 immune checkpoint negative regulators between the DDR Mut and DDR groups. ***P<0.001, ****P<0.0001. [file Image_4.tif]

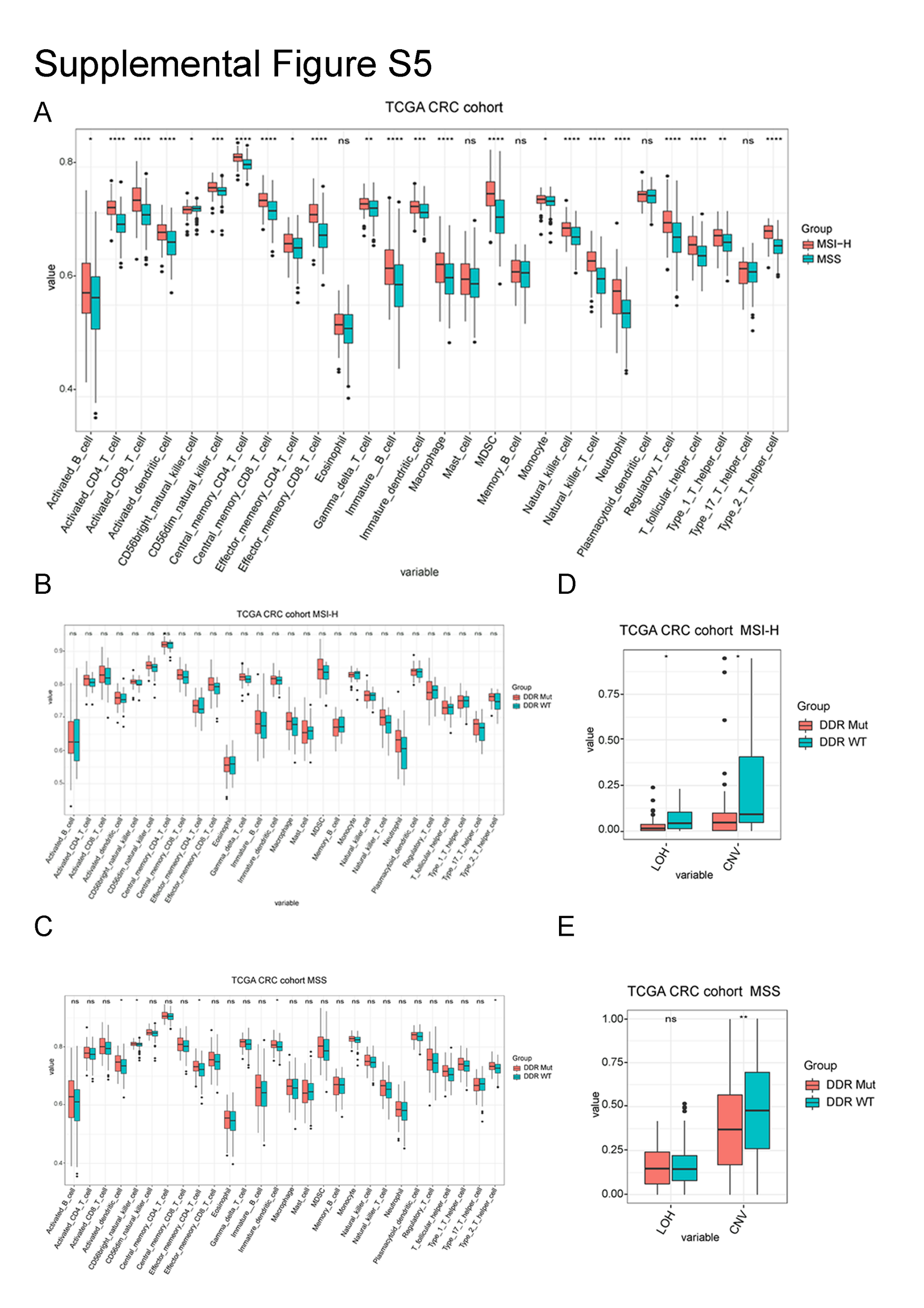

Supplement: Supplementary Figure 5 — DDR mutation improves the efficacy of ICI immunotherapy via regulation of the immune microenvironment in MSS subgroup. (A) Comparison of 28 immune cell subsets between the MSI-H and MSS groups. (B) Comparison of 28 immune cell subsets, (D) LOH and CNV between DDR Mut and WT groups in MSI-H subgroup. (C) Comparison of 28 immune cell subsets, (E) LOH and CNV between DDR Mut and WT groups in MSS subgroup. ns P>0.05, * P<0.05, ** P<0.01, ***P<0.001, ****P<0.0001. [file Image_5.tif]

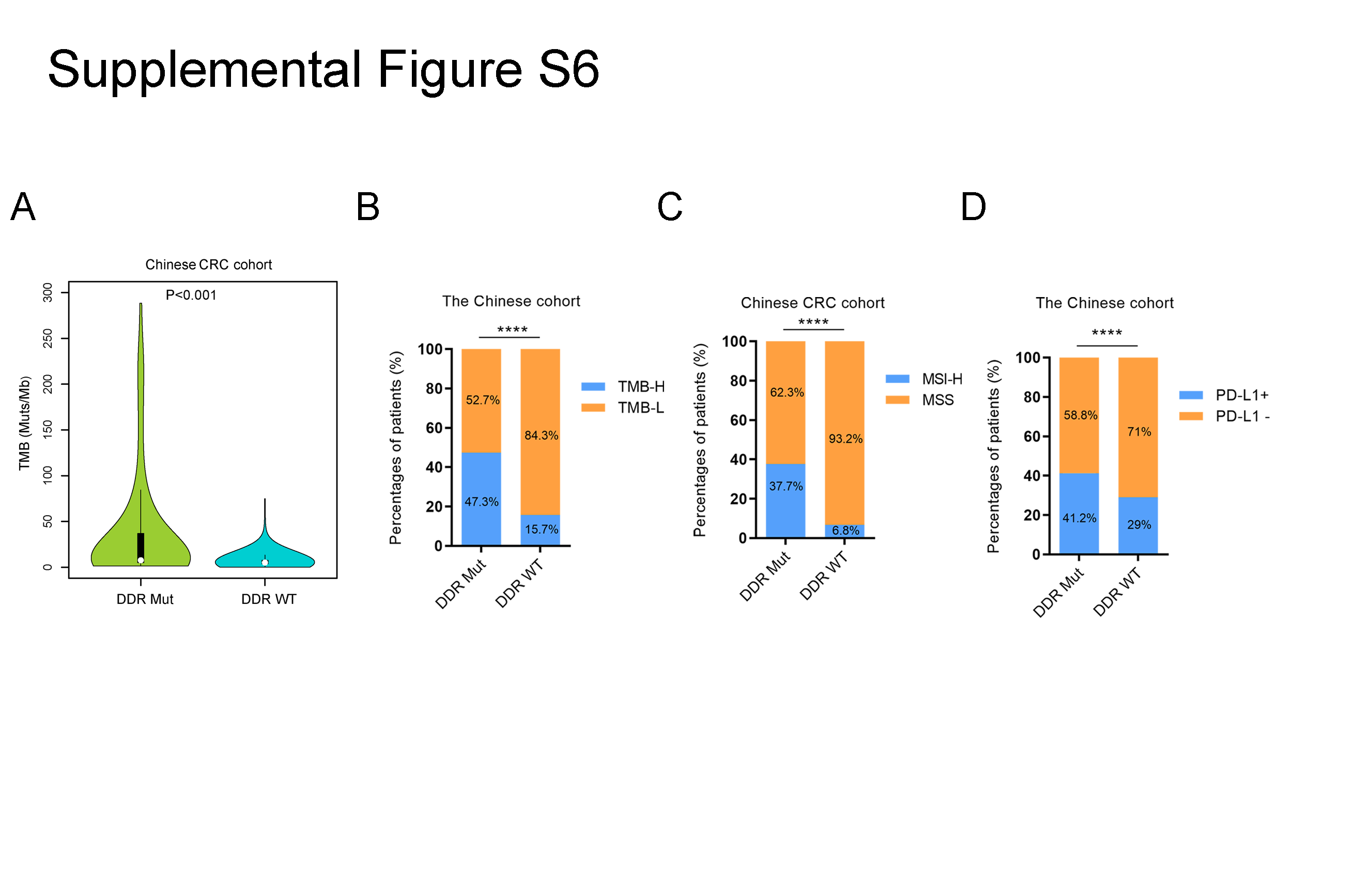

Supplement: Supplementary Figure 6 — The association between the TMB value and the incidence of DDR mutation, the MSI status and the incidence of DDR mutation, PD-L1-positivity and the incidence of DDR mutation in a Chinese CRC cohort. (A) Distribution of TMB in the DDR Mut and WT groups. (B) The proportion of TMB-High and TMB-Low in the DDR Mut and WT groups. (C) The proportion of MSI-H and MSS in the DDR Mut and WT groups. (D) The proportion of PD-L1+ and PD-L1- in the DDR Mut and WT groups. [file Image_6.tif]
