# Supplementary material for: DNA Damage Repair Gene Mutations Are Indicative of a Favorable Prognosis in Colorectal Cancer Treated With Immune Checkpoint Inhibitors
Source: Front Oncol. 2021 Feb 19;10:549777. doi: 10.3389/fonc.2020.549777 (PMC7934780; doi:10.3389/fonc.2020.549777)
Supplement: Supplementary file 7 [file DataSheet_1.docx]

**Supplemental materials**

**Method for MSI identification**

In TCGA cohort, the MSI data was obtained from [Russell Bonneville](https://pubmed.ncbi.nlm.nih.gov/?term=Bonneville+R&cauthor_id=29850653)’ publication. Using published MSI-calling software, MANTIS, [Russell Bonneville](https://pubmed.ncbi.nlm.nih.gov/?term=Bonneville+R&cauthor_id=29850653) analyzed whole-exome data from 11,139 tumor-normal pairs from The Cancer Genome Atlas and Therapeutically Applicable Research to Generate Effective Treatments projects and external data sources across 39 cancer types (1).

In Chinese cohort: the MSI and TMB of CRC patients were detected using next generation sequencing (NGS) in Genecast Biotechnology Co., Ltd. The Genecast panel was a 1.67 Mbp-sized panel covering the exon regions of 543 genes (Sup Table S1; Genecast, Wuxi, China), including major tumor-related genes and 208 microsatellite locus. The method for NGS MSI identification was developed by Genecast Biotechnology Co., Ltd. The MSI calculation pipeline was described as follow,

1. Adaptors of raw read pairs were trimmed using Trimmomatic (v0.39) . Clean reads were mapped against the human reference genome (build hg19, UCSC) using bwa-mem (v0.7.12) and sorted using SAMtools (v1.3). MarkDuplicates was performed followed by local indel realignment using GATK version v2.8. For each microsatellite locus, all spanning reads (covering at least 2bp in both 5’ and 3’ directions) were extracted from realigned BAM file. Following deduplication, the length of the mononucleotide repeat in each deduped alignment was counted and tallied by lengths.
2. Thirty healthy blood samples were evaluated the number of alleles of each observed length compared to the reference genome within each of the microsatellite locus and then the mean and SD of the number of alleles were calculated as the baseline reference value.

3**.** Experimental results were compared against baseline reference values at each locus to assess the instability of microsatellite loci. If the tally of alleles counted exceeded [mean number of alleles + (4 x SD)] the MSI stable reference value, the locus was scored as unstable, and if the tally did not exceed this value, it was scored as stable. Finally, the fraction of unstable loci out of the total number of loci analyzed was calculated for each experimental sample. The cutoff fraction of 0.3 (20%) unstable loci was set for an MSI positive result.

**The Calling Criterion for the Somatic Mutations in Chinese Cohort**

In Chinese cohort, we used VarScan2 (v2.4.2) to call somatic somatic mutations on tumor samples and matched blood samples. The following filters were applied: (i) number of mutant allele reads > 2; (ii) coverage in normal > 50 and coverage in tumor > 100; (iii) mutant allele frequency > 2%; (iv) nonsynonymous SNVs and indels; (v) located in exon regions; and (vi) allele frequency<0.5% in the exac03 database.

1. Bonneville R, Krook MA, Kautto EA, Miya J, Wing MR, Chen HZ, et al. Landscape of Microsatellite Instability Across 39 Cancer Types. *JCO Precis Oncol* (2017) 2017.
