# Supplementary material for: DNA Damage Repair Gene Mutations Are Indicative of a Favorable Prognosis in Colorectal Cancer Treated With Immune Checkpoint Inhibitors
Source: Front Oncol. 2021 Feb 19;10:549777. doi: 10.3389/fonc.2020.549777 (PMC7934780; doi:10.3389/fonc.2020.549777)
Supplement: Supplementary file 8 [file Table_1.docx]

| **Supplemental Table S1. Genecast panel gene list.** | | | | | | | | | |
| --- | --- | --- | --- | --- | --- | --- | --- | --- | --- |
| ABCA13 | ATR | CCND1 | CRLF2 | ERBB3 | FMO1 | HSPA1B | KPNA4 | MSH2 | NTRK1 |
| ABCA8 | ATRX | CCND2 | CSF1R | ERBB4 | FOLH1 | HSPA4 | KPNB1 | MSH3 | NTRK2 |
| ABCB1 | AURKA | CCND3 | CSF3R | ERCC1 | FOXL2 | HSPA5 | KRAS | MSH6 | NTRK3 |
| ABCC2 | AURKB | CCNE1 | CTCF | ERCC2 | FOXP1 | HYOU1 | LAMA3 | MTF1 | NUP85 |
| ABL1 | AXIN1 | CCR4 | CTNNB1 | ERCC4 | FRAS1 | IARS | LEPR | MTHFR | NUP93 |
| ACADSB | AXL | CD274 | CUL3 | ERG | FUBP1 | ID2 | LMO1 | MTOR | OTOS |
| ACOT13 | B2M | CD40 | CXCL8 | ERI1 | FUS | ID3 | LONRF3 | MTR | P2RY8 |
| ADAMTS6 | BAP1 | CD74 | CXCR4 | ERRFI1 | GABRP | IDH1 | LRP2 | MTRR | PALB2 |
| ADRB1 | BARD1 | CD79A | CYBA | ESR1 | GALNT14 | IDH2 | LRRC34 | MUTYH | PAPOLG |
| ADSS | BCL2 | CD79B | CYFIP1 | ETV1 | GANC | IGF1R | LYN | MYADM | PAQR8 |
| AK7 | BCL2L1 | CDA | CYLD | ETV6 | GATA1 | IGF2 | MAGOHB | MYC | PARP1 |
| AKT1 | BCOR | CDC25B | CYP19A1 | EWSR1 | GATA2 | IKBKE | MALT1 | MYCL | PAX5 |
| AKT2 | BCYRN1 | CDC73 | CYP2B6 | EXOSC8 | GATA3 | IKZF1 | MAP2K1 | MYCN | PBRM1 |
| AKT3 | BLM | CDH1 | CYP2C19 | EZH2 | GLI1 | IL7R | MAP2K2 | MYD88 | PDCD1 |
| ALG9 | BRAF | CDK12 | CYP2C8 | EZR | GMEB1 | INHBA | MAP2K4 | MYO10 | PDCD1LG2 |
| ALK | BRCA1 | CDK4 | CYP2D6 | F13A1 | GNA11 | INPP4B | MAP3K1 | NAB1 | PDE6C |
| ALOX12B | BRCA2 | CDK6 | DAXX | FAM149A | GNA13 | IPO7 | MAP3K4 | NAB2 | PDGFB |
| AMER1 | BRD4 | CDK7 | DBT | FAM153B | GNAQ | IRAK1 | MAP4K5 | NBN | PDGFRA |
| ANKRA2 | BRIP1 | CDK8 | DDR2 | FANCA | GNAS | IRF4 | MAPK1 | NCOA6 | PDGFRB |
| ANKRD46 | BRS3 | CDKL3 | DEPDC5 | FANCC | GPAT3 | IRF6 | MAPKAP1 | NDUFS1 | PDPN |
| ANO1 | BTF3 | CDKN1A | DHFR | FANCD2 | GPM6A | IRF8 | MAPKBP1 | NEO1 | PGBD1 |
| APC | BTG1 | CDKN1B | DIAPH1 | FANCG | GRIN2A | IRS2 | MARK2 | NF1 | PIGF |
| APOL2 | BTK | CDKN2A | DICER1 | FANCI | GSK3B | ITGAL | MCL1 | NF2 | PIK3C2G |
| APOPT1 | C20orf96 | CDKN2B | DIS3 | FAS | GSTA1 | JAK1 | MDM2 | NFE2L2 | PIK3CA |
| AR | C22orf23 | CDKN2C | DNMT3A | FAT1 | GSTM1 | JAK2 | MDM4 | NFKBIA | PIK3CB |
| ARAF | C2CD6 | CDO1 | DOCK11 | FBXW7 | GSTP1 | JAK3 | MED12 | NFXL1 | PIK3CG |
| ARHGAP4 | C5orf15 | CEBPA | DOT1L | FGF16 | H3F3A | JUN | MED19 | NKAP | PIK3R1 |
| ARHGAP6 | C8orf34 | CEP120 | DPYD | FGF19 | HAUS2 | KCNJ2 | MEF2B | NKX2-1 | PIK3R2 |
| ARID1A | C9orf72 | CEP290 | DROSHA | FGF3 | HAUS6 | KDM5A | MEIS1 | NLRP7 | PLCG2 |
| ARID1B | CAB39 | CHD1 | DSCAM | FGF4 | HCAR2 | KDM5C | MEN1 | NOTCH1 | PLEKHA1 |
| ARID2 | CALD1 | CHEK1 | DYNC2H1 | FGFR1 | HEY1 | KDM6A | MET | NOTCH2 | PLEKHH2 |
| ARID4A | CALM2 | CHEK2 | EGFR | FGFR2 | HGF | KDR | MIA2 | NOTCH3 | PMS2 |
| ARL6IP6 | CALR | CIC | EIF4G3 | FGFR3 | HLA-A | KEAP1 | MITF | NPM1 | PNO1 |
| ARMC5 | CARD11 | CNKSR3 | EML4 | FGFR4 | HLA-B | KIAA1210 | MLH1 | NR1I3 | POLD1 |
| ARPC2 | CASP8 | CNOT8 | EP300 | FH | HLA-C | KIF5B | MMP16 | NR4A3 | POLE |
| ASH1L | CAST | COL15A1 | EPHA3 | FLCN | HLA-DRB1 | KIR3DX1 | MMP3 | NRAS | PPARG |
| ASXL1 | CBFB | COX18 | EPHA5 | FLOT1 | HNF1A | KIT | MOV10L1 | NSD1 | PPHLN1 |
| ATIC | CBL | CPLANE1 | EPHA7 | FLT1 | HNF4A | KMT2A | MPL | NSD2 | PPP2R1A |
| ATM | CBR3 | CREBBP | EPHB1 | FLT3 | HNRNPH1 | KMT2C | MRE11 | NSD3 | PRDM1 |
| ATP9B | CBR4 | CRKL | ERBB2 | FLT4 | HRAS | KMT2D | MRPL19 | NT5C2 | PREX2 |
| PRKAR1A | RAD51C | RIPK2 | SEL1L3 | SLIT1 | SRC | TBC1D8B | TOE1 | UBE3C | ZDHHC17 |
| PRKCI | RAD51D | RNF19A | SEMA3C | SMAD2 | SS18 | TBX3 | TOP1 | UGT1A1 | ZMYM4 |
| PRKN | RAD52 | RNF43 | SETD2 | SMAD3 | STAG2 | TECPR2 | TOP2B | ULK4 | ZNF2 |
| PRPF39 | RAD54L | ROS1 | SF3B1 | SMAD4 | STARD4 | TENT5C | TP53 | UMPS | ZNF367 |
| PTCH1 | RAF1 | RPA4 | SFXN4 | SMARCA4 | STAT3 | TERT | TPH1 | UPF2 | ZNF711 |
| PTEN | RARA | RPTOR | SHROOM3 | SMARCB1 | STK11 | TET2 | TRA2A | VEGFA | ZNF805 |
| PTPN11 | RB1 | RRM1 | SIMC1 | SMO | STMN1 | TGFBR2 | TRIM24 | VHL | ZNF91 |
| PTPRJ | RBM10 | RRP1B | SIPA1L2 | SNX6 | STRBP | TMEM67 | TSC1 | VSIG10 | ZZZ3 |
| PURA | RBM27 | RUNX1 | SLC22A2 | SOCS1 | STYX | TMPRSS15 | TSC2 | WDR5 |  |
| RABGAP1L | REL | RYR2 | SLC30A5 | SOD2 | SUCLG1 | TMPRSS2 | TSHR | WT1 |  |
| RAC1 | RET | SASH1 | SLC31A1 | SOX2 | SUFU | TNFAIP3 | TSN | WWC3 |  |
| RAD21 | RFC1 | SDHA | SLC34A2 | SOX9 | SUGCT | TNFRSF14 | TXNRD1 | XPC |  |
| RAD50 | RHOT1 | SDHB | SLC7A8 | SPC24 | SYK | TNFSF13B | TYMS | XPO1 |  |
| RAD51 | RIC1 | SDHC | SLCO1B1 | SPEN | TAF15 | TNKS | U2AF1 | XRCC1 |  |
| RAD51B | RICTOR | SDHD | SLCO1B3 | SPOP | TAGAP | TNRC18 | UBE2E3 | ZBBX |  |
